# Successful Treatment of Multiple Multidrug Resistant Intracranial Tuberculomata

**DOI:** 10.1155/2016/1841529

**Published:** 2016-12-29

**Authors:** Richard P. Sullivan, Hazel F. Goldberg, Ross S. Mellick, Jeffrey J. Post

**Affiliations:** ^1^Department of Infectious Diseases, Prince of Wales Hospital, Sydney, NSW, Australia; ^2^Prince of Wales Clinical School, University of NSW, Sydney, NSW, Australia; ^3^Department of Respiratory Medicine, Prince of Wales Hospital, Sydney, NSW, Australia; ^4^Institute of Neurological Sciences, Prince of Wales Hospital, Sydney, NSW, Australia

## Abstract

A 21-year-old Bangladesh-born man presented with a month history of evolving neurological symptoms in the context of a six-month history of fever, night sweats, and axillary lymphadenopathy. He was subsequently diagnosed with multiple multidrug resistant intracranial tuberculomata and was successfully treated over two years. Intracranial multidrug resistant tuberculosis has a high mortality and successful treatment is rarely reported. Management is complex and requires consideration of the penetration and likely effect of antituberculous agents within the central nervous system. We discuss the role of various antituberculous agents, the duration of therapy, the utility of corticosteroids, the value of intrathecal and systemic therapy, and the need for rapid diagnosis.

## 1. Introduction

There are no clear guidelines on the management of central nervous system multidrug resistant tuberculosis (MDR CNS TB), which is rarely reported and is associated with a high mortality [1]. We describe a case of successful treatment of a man with multiple, multidrug resistant intracranial tuberculomata.

## 2. Case Report

**Figure 1:**
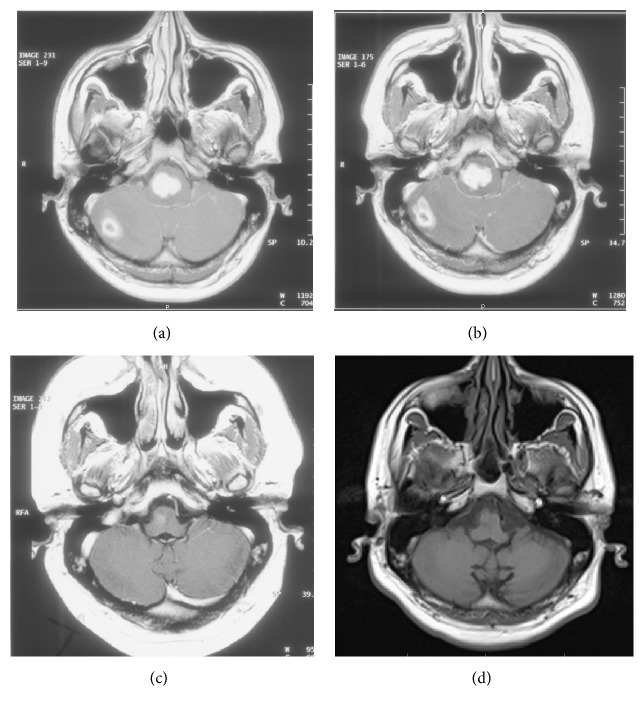
MRI scan during treatment phases. At diagnosis (a), one month after diagnosis on HREZ (b), after two months of therapy based on resistance assay (c), and after five years (d).

A previously well 21-year-old Bangladesh-born man presented with a six-month history of night sweats, fever, and axillary lymphadenopathy and a four-week history of evolving neurological symptoms including vertigo, diplopia, ataxia, left sided weakness, and right facial droop. On examination there were multiple cranial nerve palsies, reduced power in the left upper and lower limbs, and enlarged left axillary lymph nodes. Multiple enhancing lesions in the pontomedullary junction, right cerebellum, and left frontal lobe were detected on magnetic resonance imaging (MRI) (see Figure 1(a)). A left axillary mass biopsy demonstrated granulomatous lymphadenitis, with negative Truant's stain and negative mycobacterial PCR. HIV testing was negative, and a CT of the chest, abdomen, and pelvis showed isolated left axillary lymphadenopathy.

Rifampicin, isoniazid, pyrazinamide, ethambutol (HREZ), and dexamethasone were commenced. He had partial neurological improvement in left sided weakness but approximately one month later developed nausea, vomiting, dysphagia, and headache, with further reduction in power and coordination on the left. Repeat MRI showed increased oedema and size of the pontomedullary and right cerebellar lesions with hydrocephalus (see Figure 1(b)). The dexamethasone dose was increased, HREZ continued, and he improved symptomatically.* Mycobacterium tuberculosis* was isolated from the lymph node biopsy and after seven weeks of therapy provisional rifampicin and probable isoniazid resistance was identified. Therapy was changed to rifabutin, isoniazid, pyrazinamide, ethambutol, amikacin, moxifloxacin, cycloserine, and prothionamide. Final susceptibility results demonstrated resistance to isoniazid, rifampicin, rifabutin, clofazimine, and streptomycin with sensitivity to ethambutol, pyrazinamide, amikacin, capreomycin, ciprofloxacin, cycloserine, and ethionamide. The regimen was continued without rifabutin. After two weeks of inpatient treatment, he was discharged on a weaning dose of dexamethasone, with mild left facial droop and paresthesia in the left arm.

Repeat MRI approximately two months after commencement of the MDR TB regimen showed a marked reduction in the size of the right-sided pontine and cerebellar lesions (see Figure 1(c)). Dexamethasone was ceased, precipitating an adrenal crisis and immune reaction in the right middle lobe of the lung. He was recommenced on steroids, improved, and was discharged on a weaning dose of cortisone. Amikacin was ceased after twelve months while other treatment was continued for a total of two years after repeat MRI showed almost complete resolution of the lesions. He was monitored for the next five years with repeat MRI scanning showing complete resolution of the right cerebellar lesion and continued improvement in the pontomedullary lesion (see Figure 1(d)). Complications during treatment included avascular necrosis of the hip, which was managed conservatively. After seven years of follow-up, his residual symptoms included minor difficulty with left upper limb fine motor tasks and hip pain.

## 3. Discussion

This case highlights management considerations in MDR CNS TB. To our knowledge, there are only case reports of successful treatment of MDR CNS TB [2–4]. The British Infection Society recommends initial treatment with at least fluoroquinolone, pyrazinamide, ethionamide, or prothionamide and an injectable agent, while the World Health Organization recommends use of agents with good CNS penetration [5, 6]. We selected a regimen based on the likely pharmacodynamic and pharmacokinetic properties of antituberculous agents within the CNS and the susceptibility profile of the organism. Management questions remain about the optimal duration of treatment and which agents should be used to maximize effect while minimizing toxicity.

Data on the CNS penetration of second-line antituberculous agents is limited. This is an important management consideration as poor penetration and the lack of an effective immune response in the CNS can affect response [6]. Some drugs used in management of MDR CNS TB penetrate the blood-brain barrier and these include quinolones, pyrazinamide, linezolid, cycloserine, and ethionamide [5–7]. This property makes them important in treatment regimens, and they should be used in consultation with the specific susceptibility profile. We also treated with ethambutol, which penetrates the blood-brain barrier in the presence of inflammation, but not otherwise due to its high molecular weight [2, 5]. Amikacin was chosen over capreomycin as it has better cerebrospinal fluid penetration [8]. An approach which utilizes a number of these agents is recommended rather than addition of single agents to the HREZ regimen [8].

The use of isoniazid in the management of isoniazid resistant disease is controversial. One study demonstrated patients with isoniazid resistant (with or without streptomycin resistance) TB meningitis did not have different outcome to patients with sensitive TB meningitis when treated with isoniazid, rifampin, streptomycin, and pyrazinamide. Conversely, patients with MDR TB meningitis had a one hundred percent mortality [1]. The British Society Guidelines recommend use of isoniazid in isolated low-level isoniazid resistant disease [5]. The WHO recommends use of high dose isoniazid in MDR CNS TB unless there is high level isoniazid resistance [6]. Its benefits include a highly bactericidal action and good penetration to the CNS, which allow high peak concentrations in the CSF, often exceeding minimum inhibitory concentrations.

It is not known if second-line antituberculous agents are effective in the CNS at the concentrations delivered. For instance, the role of pyrazinamide is controversial, as while it penetrates the CNS well, its efficacy in this compartment has been questioned [2]. An improvement in MDR TB pulmonary disease occurred while CNS disease worsened in one case, highlighting the need to consider the CNS as a separate therapeutic environment [2]. In addition, adequate concentrations may not be reached at standard dosing. Higher doses of many of the antituberculous agents are therefore used during the initial phases of treatment [5].

It has been reported that intrathecal amikacin and levofloxacin have led to successful treatment following failure of systemic administration [2]. The mechanism of this is not entirely clear as intravenous therapy provided adequate CSF concentrations in that patient [2]. We would encourage further study on adequate dosing and on the utility and indications of both intrathecal and systemic drug administration.

Corticosteroids were initially used with transient effect on neurological signs in the absence of effective antituberculous agents, likely due to a reduction in inflammation and intracranial pressure [8]. While steroids have been shown to improve outcome in HIV negative patients with TB meningitis, there are currently no studies on tuberculoma outcomes [5].

The optimal treatment duration for MDR CNS TB is not known. Early diagnosis of MDR TB is critical in ensuring adequate treatment is given as early. New PCR methods and neuroradiological diagnostic criteria for tuberculomata may be useful, particularly assays that can detect resistance early. The sensitivity and specificity of these tests limit their utility. We searched for extraneural sites for biopsy, which allowed early commencement of treatment. However, there was a seven-week delay until drug susceptibility data was available. A reduction in these delays will ensure better outcomes. Length of treatment was largely guided by imaging, and our patient had no relapse over seven years. The optimal duration of therapy is still not known.

To our knowledge this is the first reported successful treatment of CNS MDR tuberculomata using this regimen. The regimen used was consistent with the approach of the British Infection Society and WHO guidelines and the outcomes reported in this case support the use of these approaches.
